# From Animal to Human: (Re)using Acellular Extracellular Matrices for Temporomandibular Disc Substitution

**DOI:** 10.3390/jfb13020061

**Published:** 2022-05-18

**Authors:** Daniela Trindade, Nuno Alves, Carla Moura

**Affiliations:** Centre for Rapid and Sustainable Product Development (CDRSP), Polytechnic of Leiria, 2430-028 Marinha Grande, Portugal; daniela.trindade@ipleiria.pt (D.T.); nuno.alves@ipleiria.pt (N.A.)

**Keywords:** temporomandibular disc, decellularization, extracellular matrix

## Abstract

Current treatments for temporomandibular joint (TMJ) disc dysfunctions are not fully effective and lack regenerative capacity. Therefore, the search for tissue-engineered materials for TMJ disc substitution is critical to fill this gap. Decellularization presents tremendous potential, as it is possible to obtain an extracellular matrix with an adequate biomechanical structure and biochemical components. However, its application to the TMJ disc is still in progress, since there are few studies in the literature, and those that exist have many gaps in terms of characterisation, which is decisive to ensure its success. Ultimately, we intend to emphasize the importance of the decellularization technique for the development of an engineered TMJ disc.

## 1. Introduction

It is known that the human body is a complex, well organised, and optimised “machine”. However, sometimes it fails so upon trauma, the development of curative therapies for the temporomandibular joint (TMJ) disc is of extreme importance. Since mimicking the disc is challenging due to its dense and complex extracellular matrix (ECM), we strongly believe the response to tissue repair passes through the reuse of native tissues through their decellularization. Decellularization is a technique with tremendous potential for temporomandibular disc substitution, since it allows the retention of the native ECM, which serves as a support, and biochemical signals. This is crucial for the success of the implanted disc in terms of the cells’ fate [1,2].

## 2. Why Is There a Need to Investigate TMJ Disc Substitutes?

Damage and degeneration in our body’s articulations, such as the knee, hip, shoulders, and ankles, regardless of the cause, are common, and several clinical options are already available. However, the TMJ is one of the least understood joints of our body and performs activities such as communicating, eating, and smiling [3]. Recently, Bielajew et al. reported that the knee and the TMJ have similar osteoarthritis incidence, but the latter lacks research and grant funding, cell-based therapies, and specialist doctors in the field [4]. Moreover, even one year after the end of lockdown induced by the COVID-19 pandemic, dental consultations were reduced, leading to further aggravation of TMJ dysfunctions (TMD) and bruxism [5].

When TMJ functions are compromised, strongly affecting quality of life, we are faced with TMD that affects approximately 31% of the adult/elderly and 11% of the children/adolescents [6]. In cases of TMD, the fibrocartilaginous disc that is crucial for the correct movements of the TMJ may be displaced from its native position, perforate or become thinner [7]. Conventional TMD treatments can be divided into non-invasive, minimally invasive, and invasive. In the early stage of the disease, non-invasive treatments are ideal, such as medications, physiotherapy, and/or occlusal splints. In the later stage, invasive methods such as total joint replacement or discectomy (total removal of the TMJ disc) are the best options [8]; however, the latter can lead to ankylosis [9]. Regarding minimally invasive procedures, they include intra-articular injections, arthrocentesis, and arthroscopy. Unfortunately, they lack the capacity to restore a damaged disc and are only beneficial in relieving pain [10]. In conclusion, there is an unmet treatment with a full restorative capacity and demand for such a treatment should continue.

## 3. Methodology

The search for articles in the area was carried out in PubMed and b-on with the keywords “temporomandibular joint”, “decellularized” and/or “extracellular matrix”. The selection criteria were original research that performed decellularization methods. Fulfilling these, 9 articles were found. The remaining cited articles were reviews that were considered relevant to the present study.

## 4. A Decellularized TMJ Disc Is Not Yet Achieved, but It Is Achievable

The decellularization technique has been progressively explored and investigated over the years [2]. With it, it is possible to remove the cellular content that leads to the transmission of infection and diseases by applying chemical, enzymatic, and/or physical methods, maintaining an undamaged ECM structure and the biochemical components such as proteins (collagen and elastin), cell adhesion proteins (fibronectin and laminin), glycosaminoglycans (GAGs), and growth factors [11,12]. These acellular tissues are ideal for load-bearing or high ECM content tissues, resembling the characteristics and properties of the native one [13]. Different commercial products are available from xenogeneic tissues, e.g., porcine dermis or porcine urinary bladder for soft tissues [14]. Tissue-engineered decellularized ECM approaches could help in search of an engineered disc. However, its application to the TMJ disc is still in early stages.

The first use of decellularized tissues for tissue engineering of the TMJ disc was powdered porcine urinary bladder ECM encapsulated within sheets of the same material. Despite the fact that in vivo implantation in dogs led to proper tissue formation, it is necessary to perform an analysis of the remaining bone structure [15,16].

Regarding studies that evaluate methods for TMJ disc decellularization, the first attempt was achieved by Lumpkins et al., where three different reagents were tested on porcine discs: 1% (*w*/*v*) sodium dodecyl sulfate (SDS), 1% Triton X-100 and 25% acetone/75% ethanol (vol.%) [17]. The histological results demonstrated complete cell removal from all decellularizing agents. As for mechanical properties, Triton X-100 resulted in a softer disc, as energy dissipation decreased, while acetone/ethanol made the disc stiffer and dehydrated. It was concluded that SDS is a preferred reagent because its mechanical properties have been maintained, although the collagen fibres have become compressed.

Taking into consideration this latter article, different authors tested other concentrations of SDS and other reagents to optimize the decellularization protocol. Matuska et al. investigated the decellularization of the TMJ disc as well as its retrodiscal tissue [18]. The retrodiscal tissue of the porcine disc has a higher lipidic content than that of the human disc, the combination of 0.1% SDS and 2:1 solution of chloroform/methanol was shown to be effective in removing the cellular and lipid content but the conservation of the biochemical components were not evaluated. Juran et al. addressed the problem of collagen fibre compaction by combining 1% SDS with freeze drying and rehydration of the disc, which at the same time removed the cellular content [19]. Despite this, the disc’s final mechanical properties were not assessed. Artificial porosity was also implemented with micropatterning, leading to cell integration and remodelling. This same technique was also applied by Matuska and McFetridge, but in vitro assays were not performed. In this study, a decellularization of 0.1% SDS was applied, and cellular content was removed but some collagen was lost [20]. In terms of sterilization, three methods have been investigated on TMJ discs: gamma irradiation, ethylene oxide, and peracetic acid. Although the latter led to enhanced cell attachment, all methods led to decreased compressive instantaneous moduli [21].

Although some research has been done on disc decellularization, it still has some limitations. The present articles fail to make a complete characterisation of the disc, including biochemical quantification and biomechanical properties. Furthermore, the referred above articles only investigated chemical agents, while the decellularization technique can also be applied with several enzymatic and physical agents, as well by different techniques [22]. A more complete decellularization protocol was investigated and the processing of the disc ECM into an injectable hydrogel was carried out. A combination of freeze-thaw, 1% Triton X-100, hypotonic Tris–hydrochloric acid buffer, trypsin, and nucleases was applied, and an acellular hydrogel was obtained with good injectability; however, GAG content decreased and in vivo inflammation occurred [23]. This same decellularization and post-processing technique was implemented and combined with polycaprolactone/polyurethane scaffolds coated with polydopamine. Despite fibrogenesis and chondrogenesis in vivo, the degradation and mechanical behaviour still needs improvements between the natural and synthetic materials [24].

The use of decellularized matrices for complex TMJ disc regeneration appears as a promising option but is still in its infancy. A large part of the work is focused on finding the optimal decellularization protocol. However, this has not yet been achieved as most studies do not perform an adequate characterisation, that is of paramount importance for finding a disc substitute. Within the characterisations, it is crucial to confirm that (i) the cellular content has been removed; (ii) the structure and content of collagen and GAG has been maintained, as they are the main constituents of the disc; and (iii) the viscoelastic behaviour has not been altered, as the disc is subjected to different mechanical forces due to its translation and rotation movements. Regarding this last point, the authors believe that the first step towards unravelling the complexity of the disc’s viscoelastic behaviour should be through the use of numerical models such as non-linear or quasi-linear models [25,26,27]. This will enhance the study of bioengineered discs, such as those obtained by decellularization, since a better understanding of the load distribution in TMJ will be performed. Ultimately, it will reduce laborious and complex laboratory studies as well as reduce the ethical problems with in vivo studies.

## 5. Future Outlook

Treatments for TMJ disorders are far from being fully effective in the long term. The search for TE solutions for the TMJ disc is an approach that is fully accepted by the scientific community but still has many gaps. In regard to decellularization, which the authors believe is a step forward from commonly used materials, the most effective method has yet to be found. This should be the first step for researchers in this area. When this is achieved, several treatments might be idealized, such as the use of the disc (recellularized or not) for a total replacement, or to solubilise the ECM to be used in minimally invasive treatments and, consequently, assist in the regeneration of damaged TMJ discs.
